# The case for evaluation of double mutant heat-labile toxin as an adjuvant to improve oral cholera vaccine immunogenicity

**DOI:** 10.3389/fimmu.2026.1805998

**Published:** 2026-04-20

**Authors:** A. Louis Bourgeois, Daniel A. Scott, Shahida Baqar, David R. Tribble, Ann-Mari Svennerholm, Richard I. Walker

**Affiliations:** 1 Enteric Diseases Program, Naval Medical Research Institute (NMRI), Bethesda, MD, United States; 2 Center for Immunization Research, Johns Hopkins Bloomberg School of Public Health, Baltimore, MD, United States; 3 National Institute of Allergy and Infectious Diseases, National Institutes of Health, Bethesda, MD, United States; 4 Infectious Disease Clinical Research Program, Department of Preventive Medicine and Biostatistics, Uniformed Services University of the Health Sciences, Bethesda, MD, United States; 5 Department of Microbiology and Immunology, University of Gothenburg, Gothenburg, Sweden; 6 Center for Vaccine Innovation and Access, PATH, Washington, DC, United States

**Keywords:** cholera vaccines, enterotoxigenic *Escherichia coli* vaccine, enterotoxin adjuvant, mucosal immunity, oral vaccines

## Abstract

Cholera has re-emerged as a major global public health threat. Orally administered attenuated or inactivated vaccines offer protection against enteric pathogens such as *Vibrio cholerae*, and several World Health Organization-prequalified oral cholera vaccines are used globally as part of international cholera prevention and control measures. However, vaccine effectiveness, particularly in young children in low- and middle-income countries, is often lower than in adults, leaving this vulnerable age group at greater risk of cholera. The heat-labile toxin (LT) of enterotoxigenic *Escherichia coli* (ETEC) has strong mucosal adjuvant properties, and in detoxified form (double-mutant LT), it has been shown to safely improve immune responses to protein and somatic lipopolysaccharide antigens in children in Bangladesh who received the oral inactivated whole-cell enterotoxigenic ETEC vaccine ETVAX. Subsequent field trials of ETVAX in children in Zambia and The Gambia have also further demonstrated safety and immunogenicity in this difficult-to-immunize age group. Current concerns about reduced effectiveness of oral cholera vaccines in children led us to re-examine data from an earlier unpublished study conducted to discern for the first time, the adjuvant effects of LT on an orally administered vaccine in humans. The study showed that native LT improved immune responses to a cholera vaccine in adult volunteers. More recent work with double-mutant LT administered with the ETEC whole-cell vaccine ETVAX supports the findings reported here with the oral cholera vaccine Dukoral. These data suggest that modern attenuated versions of native LT, such as double-mutant LT, should be evaluated for improving immune responses to cholera vaccines.

## Introduction

Cholera has re-emerged as a major global public health threat because it has high epidemic potential and can be fatal within hours of onset in both adults and young children if not treated properly (1–5). In 2023, the World Health Organization estimated that one billion people in 43 nations were at risk of cholera outbreaks, and in 2024, across 33 countries in five World Health Organization regions, approximately 734,000 new cholera cases and 5,162 deaths were reported (6–8). Africa, in particular, has experienced an exponential increase in cholera cases since 2022, with a 37% increase in the number of cases during the 2022–2023 period and a 71% increase in cholera-related deaths during the 2023–2024 period (6–8).

Cholera vaccines provide an example of the challenges in immunizing children and highlight the need to develop more effective immunization strategies in the future. Infants and children remain the most vulnerable to more severe forms of enteric infections (3–5), making more effective vaccination critically important for the prevention and control of potentially life-threatening enteric illness in this high-risk age group. Inactivated orally administered whole-cell cholera vaccines (OCVs) now in use are generally safe and effective in older age groups. However, current formulations have reduced efficacy in children younger than five years of age (9–14), and this age-dependent reduction in protective efficacy was even more apparent when a single-dose OCV immunization regimen was evaluated (10, 11, 14). Consequently, further research is needed to develop more immunogenic OCV formulations that provide improved protection against cholera in both high-risk adults and children living in cholera-endemic areas (12–14).

Orally administered vaccines, such as OCVs, offer a practical and effective strategy for achieving mucosal immunization. Given the number of pathogens capable of causing enteric diseases and the increasingly crowded infant immunization schedule, the development and introduction of combined or co-administered oral vaccines could have a significant positive impact in addressing this major public health threat. Oral vaccines consisting of attenuated or inactivated whole pathogens, which have relatively simple manufacturing requirements, can present a complex antigenic repertoire capable of inducing systemic and mucosal immune responses when administered orally. These characteristics have led to the successful use of oral vaccines against rotavirus, polio, and cholera. Unfortunately, oral vaccination has demonstrated limited effectiveness in young children living in low- and middle-income countries compared with adults (9–11). This situation may be changing due to the availability of a mutant form of the heat-labile toxin (LT) of enterotoxigenic *Escherichia coli* (ETEC), which is a potent mucosal adjuvant that can be administered with vaccines to enhance the immune responses to both protein and lipopolysaccharide (LPS) antigens (15–20).

## Oral cholera vaccine and native heat-labile toxin clinical trial

Naturally acquired cholera induces protection that is characterized by persistent B- and T-cell memory to both toxin and O1 lipopolysaccharide (LPS) antigens. Protection associated with OCVs tends to be relatively short-lived and is characterized by weaker systemic and mucosal responses to the O1 LPS antigen and by only transient B- and T-cell memory (14, 21, 22). To improve OCV efficacy, particularly in high-risk young children, vaccine strategies need to be considered to address these immunological challenges. The evidence presented in this report suggests that the mucosal adjuvant properties of native heat-labile toxin (nLT) or its attenuated derivatives may offer a strategy to improve the effectiveness of OCVs. Field data have also shown that immune responses to cholera antigens are enhanced in cases when enterotoxigenic ETEC is present as a coinfection, suggesting a possible adjuvant role for nLT (23). Preclinical studies in the early 1990s demonstrated that nLT improved immune responses to orally administered bacterial vaccines (24). At the time of the early proof-of-concept preclinical study by Rollwagen and colleagues (24), the licensed cholera vaccine Dukoral, an inactivated whole-cell vaccine supplemented with a recombinant form of the cholera toxin B subunit (WC/rBS), was available to assess whether the adjuvant’s benefit could also be demonstrated in humans. This led to the first, as yet unpublished, clinical trial of an oral vaccine adjuvanted with nLT, which was conducted in 1995 and 1996.

### Methods summary

This clinical trial was conducted using current Good Manufacturing Practice-grade native heat-labile toxin (nLT), purified by the method of Clements and Finkelstein (25), and was provided by the Swiss Serum Vaccine Institute, Berne, Switzerland. The commercial whole-cell/recombinant B subunit (WC/rBS) vaccine (Dukoral^®^) was provided by the Swedish National Bacteriology Laboratory, Stockholm, Sweden. Each dose of the WC/rBS vaccine contained 1 mg of recombinant cholera toxin B subunit (rCTB) (i.e., rBS) and 2.5 x 10^10^ whole cells of each of the following *Vibrio cholerae* O1 strains: heat-killed classical Inaba (strain Cairo 48), heat-killed classical Ogawa (strain Cairo 50), formalin-killed El Tor Inaba (strain Phil 6973), and formalin-killed classical Ogawa (strain Cairo 50). Subjects fasted for 90 minutes before receiving nLT, WC/rBS, and WC/rBS plus nLT in bicarbonate buffer to neutralize gastric acidity (26).

Subjects participating (n = 63) in the WC/rBS trial were healthy adult military or civilian personnel 18–50 years of age with no history of cholera vaccination or traveler’s diarrhea while traveling to a developing country within the previous five years. A unique feature of this study design was that it included groups of subjects who received a two-dose regimen (days 0 and 14) of WC/rBS vaccine alone or supplemented with 0.5, 2.5, 5, 10, 15, or 25 µg of nLT. In addition, a subgroup of subjects was included who were initially immunized with two doses of nLT on days 0 and 14 and subsequently received WC/rBS supplemented with comparable doses of nLT approximately 30 days later. This subgroup was included to assess whether prior exposure to nLT might blunt or diminish its adjuvant effect when co-administered with a vaccine.

The majority of subjects in the trial were dosed and evaluated for vaccine-adjuvant reactogenicity as inpatients (n = 41). Thirty-seven of these subjects received all scheduled vaccinations and completed follow-up, and these subjects are included in the immunological assessment details in this perspective. An additional 22 subjects received the commercial WC/rBS vaccine alone as outpatients and were also included in the immunological assessments. Vaccine-induced serum and mucosal immunogenicity was evaluated by assessing serum immunoglobulin A (IgA) and G (IgG) antibody responses and IgA and IgG antibody-secreting cell (ASC) responses after dosing with rCTB. ASC and serum antibody responses to Inaba O1 lipopolysaccharide (LPS), as well as vibriocidal antibody responses, were also evaluated after dosing. All immunological assays employed were previously published (26, 27). The IgA ASC assay used in this study served as a surrogate measure of vaccine mucosal immunogenicity (28, 29). For immunological assessments, results from subjects receiving WC/rBS with 0.5–5 µg of nLT and those receiving WC/rBS with 10–25 µg of nLT were analyzed separately and compared with those from subjects receiving WC/rBS alone. This approach was taken because of the limited number of subjects in the various nLT dosing groups and to assess whether increasing doses resulted in higher response frequencies.

### Safety

Subjects receiving the vaccine alone reported no significant vaccine-associated side effects. However, nLT was associated with transient gastrointestinal reactogenicity (primarily diarrhea), particularly at doses greater than 5µg (12.5% of subjects developed diarrhea), and this reactogenicity increased when nLT was combined with the vaccine (62.5%; data not shown).

### Antibody-secreting cell and serological responses

The standard two-dose regimen of vaccine alone induced anti-rCTB-specific IgA and IgG ASC responses and serum IgA and IgG responses (**Table 1**). ASC responses (≥5 ASC per 10^6^ peripheral blood mononuclear cells after immunization) and seroconversion (≥ 4-fold rise in antigen-specific serum antibody titer from baseline) for both antibody isotypes to rCTB following the primary immunization series ranged between 70% and 89%, respectively, for both IgA and IgG in both assays. IgA and IgG ASC response frequencies to rCTB were not enhanced when the vaccine was supplemented with nLT or when subjects were exposed to nLT before vaccination. However, geometric mean fold increases in anti-rCTB IgA antibody responses were significantly higher in subjects receiving the vaccine with nLT, particularly among subjects receiving the vaccine with 0.5 – 5.0 µg of nLT (**Table 1**).

**Table 1:** Comparative serum immunoglobulin A (IgA) and IgA antibody-secreting cell (ASC) responses in adults given Dukoral (WC/rBS) alone or supplemented with different doses of native heat-labile enterotoxin (nLT) (with reference to IgG responses in the footnote).

nLT or WC/rBS		Number of subjects	Anti-O1 LPS IgA-ASC^3^		Anti-CTB IgA-ASC		Anti-O1 LPS serum IgA^3^			Anti-CTB serum IgA		
nLT (µg)	WC/rBS		GeoMean	% Responder^1^ (95% CI)	GeoMean	% Responder^1^	GMT	GMFI	% Responder^2^ (95% CI)	GMT	GMFI	% Responder^2^ 95% CI
0	WC/rBS	27	0.33	3.7 (0.1 - 19.1)	60.9	88.9	77.2	1.2	7.4 (0.9 - 24.9)	120.2	3.6	74.1 (53.7 - 88.9)
0.5–5	WC/rBS	15	46.2	46.7^4***^ (21.3 - 73.4)	59.2	73.3	110.5	2.2	40.0^**^ (16.3 - 67.7)	178.7	17.5^**^	80.0 (51.9 - 95.7)
10–25	WC/rBS	17	27.7	70.6^****^ (44.0 - 90.0)	63.6	88.2	115.5	2.6	35.2^*^ (12.2-60.7)	151.7	8.6^**^	70.6 (44.4 - 89.7)
Prior nLT exposure												
0.5–5	WC/rBS	5	8.2	40.0^*^ (5.3 - 85.3)	44.4	80.0	139.3	3.0	60.0^**^ (14.7 - 94.7)	178.7	18.4^**^	80.0 (28.4 - 99.5)
10–25	WC/rBS	10	21.6	70.0^***^ (34.6 - 93.0)	30.9	80.0	85.2	2.9	50.0^**^ (18.7 - 81.3)	138.2	6.4*	60.0 (26.2 - 87.8)

^1^ Responders are defined as ≥ 5 IgA-secreting cells per 10^6^ peripheral blood mononuclear cells (PBMCs); GeoMean = geometric mean number of antigen-specific IgA-secreting cells per 10^6^ PBMCs.

^2^ Responders are defined as a ≥ 4-fold rise in serum IgA titer post-immunization over baseline. GMT = geometric mean titer; GMFI = geometric mean fold increase over baseline.

^3^ ASC and serum responses were measured using Inaba O1 LPS and recombinant cholera toxin B subunit (rCTB). Anti-rCTB ASC and serum IgG response frequencies (73%–87%) were comparable to those for IgA and were not enhanced by nLT. IgG ASC and serum response frequencies to Inaba O1 LPS (30%–47%), as well as the magnitude of IgG responses to both rCTB and Inaba O1 LPS, were significantly enhanced by nLT supplementation (p < 0.05). IgG responses to both rCTB and O1 LPS were not significantly diminished by prior nLT exposure (data not shown).

^4^ Statistical comparisons were conducted using Fisher’s Exact Test for specified groups receiving nLT-supplemented vaccine vs. subjects receiving vaccine alone; T-tests were used to compare geometric means and fold increases: ^*^ Not statistically significant, p > 0.05; statistically significant at the range of p-values indicated, ^**^ p ≤ 0.05; ^***^ p ≤ 0.01; ^****^ p ≤ 0.001. For point estimates of percent responders and geometric mean titers and geometric mean fold increases, 95% confidence interval (95% CI) were calculated for all but only those for percent responders are shown above in an effort to simplify the data presentation.

In contrast, nLT substantially increased O1 LPS IgA ASC responses (46%–76% responders; p ≤ 0.01) and serum IgA responses to O1 LPS (35%–40% responders; p ≤ 0.05) when added to the WC/rBS vaccine (**Table 1**). Compared with vaccine alone, O1 LPS IgG ASC and serum IgG response frequencies were 33% and 46%, respectively, versus 0% and 7.4% in subjects receiving WC/rBS alone (p < 0.05) (see **Table 1** legend). Except for the O1 LPS IgA ASC response, higher doses of nLT did not increase the frequency of LPS responders in a dose-dependent manner. Administration of WC/rBS with nLT also significantly increased the magnitude of IgA and IgG ASC responses to O1 LPS (p < 0.001). As with ASC and serum responses to rCTB, responses to O1 LPS were not significantly diminished by prior exposure to nLT. These results are encouraging because supplementing WC/rBS with nLT improved its ability to induce serum and mucosal immune responses to Inaba O1 LPS, an antigen associated with broader cholera protective immunity in the field against both Inaba and Ogawa serotypes (30, 31). This improvement in the vaccine’s ability to induce O1 LPS responses with nLT, along with its well-established ability to induce strong anti-CTB responses (14, 26, 27), would likely serve to further improve overall protective efficacy.

### Vibriocidal responses

Vibriocidal antibody responses following vaccination with WC/rBS alone were modest (29.6% with ≥ 4-fold rises) and comparable to other studies conducted with this vaccine in a similar time frame in the US and Thai subjects and among US adults with a similar age and immunological background to the volunteers in this study (26, 32) (**Table 2**). Vibriocidal response frequencies were consistently higher among subjects receiving the nLT-supplemented vaccine than among subjects receiving the vaccine alone (40%–63%), but these increases were not statistically significant. Overall, vibriocidal responses were slightly higher among the relatively small number of subjects with pre-immunization titers of < 40 (38.5% with vaccine alone and 54%–60% with the nLT-supplemented vaccine), but these responses were not significantly different from those observed in the full study population.

**Table 2:** Comparative functional vibriocidal antibody responses in adults given Dukoral (WC/rBS) alone or supplemented with different doses of the native heat-labile enterotoxin (nLT).

Groups	All subjects					Subjects with baseline titers < 40				
WC/rBS with nLT (µg)	Number of subjects	Vibriocidal antibody (GMT)^2^		GMFI^2^	% Responders^1^ (95% CI)	Number of subjects	Vibriocidal antibody (GMT)^2^		GMFI^2^	% Responders^1^ (95% CI)
		Pre-vaccination	Post-vaccination				Pre-vaccination	Post-vaccination		
0	27	33.4	70.2	2.2	29.6 (18.1 - 61.6)	13	5.0	16.2	3.2	38.5 (13.9 - 68.4)
0.5–5	15	23.2	105.6	4.8^3^*	40.0^*^ (16.3 - 67.7)	10	5.8	50.3	8.7^**^	60.0^*^ (26.2 - 87.8)
10–25	16	16.2	88.5	5.5^**^	62.5^*^ (35.4 - 84.8)	13	5.9	46.7	7.9^**^	53.8^*^ (25.1 - 80.8)
Prior nLT exposure										
0.5–5	5	29.9	183.8	6.1^*^	40.0^*^ (5.2 - 85.3)	4	7.2	100.3	13.9^*^	75.0^*^ (14.7 – 94.7)
10–25	10	12.9	42.6	4.5^*^	60.0^*^ (26.2 - 87.8)	7	5.0	14.9	3.0*	28.6 (2.5 - 55.6)

^1^ A vibriocidal antibody responder is defined as a ≥ 4-fold rise in titer post-immunization over the baseline titer. Results are shown for all subjects and for subjects with baseline titers < 40 because prior studies have shown it may be more difficult to observe ≥ 4-fold rises in subjects with higher baseline titers prior to immunization (26, 32). Note: Subjects were not randomized based on baseline vibriocidal titers prior to immunization.

^2^ GMT = geometric mean titer; GMFI = geometric mean fold increase in titer over baseline.

^3^Statistical comparisons were conducted using Fisher’s Exact Test for specified groups receiving nLT-supplemented vaccine vs. control groups receiving vaccine alone. T-test were used to compare geometric means and fold increases: ^*^ Not statistically significant, p > 0.05; ^**^ statistically significant, p ≤ 0.05. For point estimates of percent responders and geometric mean titers and geometric mean fold increases, 95% confidence interval (95% CI) were calculated for all but only those for percent responders are shown above in an effort to simplify the data presentation.

The geometric mean fold increase (GMFI) from baseline in vibriocidal antibodies ranged from 2.2 to 3.2 in subjects receiving vaccine alone and from 4.8 to 8.7 in subjects receiving the nLT-supplemented vaccine (**Table 2**). Modest increases in the frequency and magnitude of vibriocidal responses were most apparent in subjects receiving WC/rBS supplemented with nLT, particularly among subjects with baseline titers < 40 (GMFI range, 7.9–8.7) (**Table 2**). The increase in response magnitude was not significantly diminished by prior exposure to nLT in subjects receiving the WC/rBS with 0.5 – 5 µg of nLT (responses frequencies also were not diminished in this group) (**Table 2**). In contrast, the vibriocidal response did appear to be reduced in subjects with prior exposure receiving WC/rBS with higher doses of nLT (**Table 2**). In addition to the increases in mucosal and serum O1 LPS responses described earlier, the higher levels of vibriocidal antibodies observed in subjects receiving the nLT-supplemented vaccine would be expected to improve vaccine efficacy because both are immune markers associated with cholera protective immunity (14, 33).

## Development of an attenuated form of heat-labile toxin

Native heat-labile toxin (nLT) promotes immune responses to antigens that are co-delivered after admixing nLT and the antigen in aqueous buffer (15, 16). However, nLT-associated oral and nasal toxicity (15) necessitated detoxification in order to use this molecule safely as a vaccine adjuvant.

nLT has an AB5 structure composed of an enzymatically active A subunit, noncovalently associated with five B-subunit monomers. A detoxified form of this protein has been achieved by the substitution of two amino acids in the toxic A subunit without loss of adjuvanticity. Glycine was used to replace arginine at amino acid 192 (R192G, or mutant LT), and alanine was substituted for leucine at amino acid 211 (L211A). LTR192G/L211A is termed double-mutant LT, or dmLT (15, 16). dmLT has significantly reduced enterotoxicity compared to nLT while retaining the adjuvant properties of the nLT (15, 16, 34).

dmLT promotes antigen-specific responses, including Th1, Th2, Th17, cytotoxic T lymphocytes, and antibodies. It augments antigen-specific IgA antibodies and long-lasting memory to co-administered bacterial and viral antigens (15, 16). Both preclinical and clinical data indicate that dmLT is a safe and effective adjuvant that, in appropriate formulations, can enhance protective immunity to vaccine antigens (15, 16). For cholera, preclinical studies have shown that co-administration of dmLT with candidate parenteral conjugate cholera vaccines can significantly enhance responses to both O1 LPS and protein antigens, as well as vibriocidal responses (35, 36). However, dmLT has not been directly tested with oral cholera vaccines in clinical trials.

## Enterotoxigenic *Escherichia coli* vaccine experience with double-mutant heat-labile toxin: ETVAX

ETVAX is an oral enterotoxigenic *Escherichia coli* (ETEC) vaccine containing inactivated *E. coli* strains overexpressing the major colonization factor antigens CFA/I, CS3, CS5, and CS6, which mediate adherence at significantly higher levels than in naturally occurring strains (37–41). This vaccine is administered together with the LT-like toxoid LCTBA, which is based on seven amino acid substitutions in the cholera toxin B subunit (42). This hybrid toxoid ensures optimal immune responses to both cholera toxin and ETEC enterotoxins. To further enhance vaccine immunogenicity, ETVAX was combined with the dmLT adjuvant. The safety and immunogenicity of ETVAX were assessed in young children and infants in Bangladesh and represented the first report of a vaccine administered with the dmLT adjuvant in this population in a lower-middle-income country (38, 39). Subsequent studies in Zambia and The Gambia have further confirmed the safety and immunogenicity of the dmLT-adjuvanted ETVAX vaccine, and protective efficacy was also recently demonstrated in The Gambia (43–45).

Following ETVAX immunization supplemented with dmLT, IgA antibody in lymphocyte secretions (ALS) responses, reflecting ASC responses, were detected against all primary vaccine antigens in most participants in the two older age groups (1–5 years) in Bangladesh, whereas these responses were less frequent and of lower magnitude in infants aged 6–11 months than in older children (39, 40). However, the addition of dmLT to ETVAX enhanced the magnitude, breadth and kinetics of intestinal (fecal) immune responses in infants after the first dose of the vaccine. Out of 139 vaccinated infants aged 6–11 months, 78 (56%) developed significantly higher mucosal responses against at least three of the five vaccine antigens after receiving dmLT, compared with 14 of 49 (29%) infants given placebo (37, 39, 40). Importantly, intestinal immune responses against the O78 lipopolysaccharide antigen, which is expressed by three of the four ETVAX vaccine strains, were significantly enhanced by the addition of dmLT in Bangladeshi infants (46). The inclusion of dmLT in the ETVAX vaccine formulation improved expression of B-cell memory markers and T-cell responses to key ETEC antigens (47, 48).

## Concluding remarks

The studies described here using an inactivated whole-cell cholera vaccine show that the adjuvant properties of nLT can significantly enhance immune responses to this vaccine. The data presented on the use of dmLT with ETVAX also support the potential benefit of this detoxified adjuvant for OCVs. Although dmLT is actively being developed as a component of the ETEC vaccine ETVAX, a similar effort is urgently needed for OCVs.

The data obtained suggest that the enhanced immune responses observed may contribute to improved protection with OCVs. Supplementation of WC/rBS with nLT resulted in broader mucosal and serum vaccine responses. Strong responses to CTB were maintained, and responses to O1 LPS were substantially enhanced. Vibriocidal responses also tended to be improved when the vaccine was administered with nLT. Thus, immune markers that have been shown to be potential predictors of cholera protective immunity were maintained or improved among subjects receiving WC/rBS supplemented with nLT (14, 33).

It is important to improve O1 LPS responses to OCVs, particularly to the O-specific polysaccharide (OSP) portion of the LPS antigen in younger age groups. Memory induction is important for longer-term protection. OSP responses were not determined in the clinical trial reported here, but the improved O1 LPS responses observed are encouraging. The findings in this small nLT pilot study need to be confirmed with dmLT in a larger study including younger age groups, but these observations help reduce uncertainty for future studies. In addition, the finding that dmLT enhanced O78 LPS responses in younger age groups in Bangladesh given ETVAX helps reduce uncertainty for follow-on studies (40). Memory cells were not assessed in this study, but results from ETVAX with dmLT in Bangladesh indicated that dmLT may enhance both B-cell and T-cell memory to co-administered antigens (47, 48). Observations from studies with ETVAX and dmLT do not guarantee the same positive effects on OCVs, but these supportive ETVAX observations suggest that follow-on studies of OCVs administered with dmLT may demonstrate similar benefits.

Studies conducted with a live attenuated ETEC vaccine, ACE 527, suggest that dmLT could also improve the immunogenicity and efficacy of live attenuated cholera vaccines, such as CVD 103-HgR or PanChol, in endemic settings where CVD 103-HgR has lacked protective efficacy in both adults and children despite being highly protective in controlled human infection model studies conducted in the US (49–51). When administered at a non-reactogenic dose to immunize volunteers, ACE 527 without dmLT did not protect against a subsequent ETEC challenge, but significant efficacy was observed in subjects receiving dmLT with the vaccine (52).

The availability of dmLT should enable work to proceed safely with OCVs to determine vaccine benefit, even when administered as a single dose, which may help expand global availability of these vaccines, a longstanding public health concern (53). In addition, this work should be designed to assess whether dmLT can provide stronger mucosal and memory responses to vaccines in infants and children, as observed in Bangladeshi infants and children who received ETVAX with dmLT. With dmLT, it may be possible to lower the age limit for OCV administration to 6 months of age, as has been accomplished with ETVAX (38). Efforts are needed to determine how the effectiveness of cholera vaccines can be improved through the use of dmLT with effective adjuvanticity as was seen with the toxic adjuvant, nLT, but with reduced toxicity.

## Data Availability

The original contributions presented in this study are included in the article. Further inquiries can be directed to the corresponding authors. Source data from the referenced studies are archived at the Naval Medical Research Command, Forest Glen, MD (WC/rBS with nLT study), or at PATH, Washington, DC (ETVAX- and ACE 527-related studies). The clinical study reports from these trials were filed with the US Food and Drug Administration under BB-INDs 5555, 16514, and 13982, and the more recent ETVAX and ACE 527 trials were registered with US, European, and African regulatory authorities (ETVAX: NCT02531802; NCT03729219; EudraCT2016-002690-35; PACTR2011905764389804; PACTR20201081021852; ACE527: NCT01739231).

## References

[1] World Health Organization . Cholera (2024). Available online at: https://www.who.int/news-room/fact-sheets/detail/cholera (Accessed January 26, 2026).

[2] Global Task Force on Cholera Control . Ending cholera: a global roadmap to 2030 (2017). Available online at: https://www.gtfcc.org/wp-content/uploads/2020/09/ending-cholera-a-global-roadmap-to-2030.pdf (Accessed January 26, 2026).

[3] Tauheed I Ahmed A Akter A Firoj MG Ahmmed F Rahman SIA . A snap-shot of a diarrheal epidemic in Dhaka due to enterotoxigenic Escherichia coli and Vibrio cholerae O1 in 2023. Front Public Health. (2023) 11:1132927. doi: 10.3389/fpubh.2023.1132927. PMID: 37124777 PMC10140589

[4] Harris JB Qadri F Ryan ET Calderwood SB . Cholera. Lancet. (2012) 379:2466–76. doi: 10.1016/S0140-6736(12)60436-X. PMID: 22748592 PMC3761070

[5] Jones A Ahmed SM Platts-Mills JA Kotloff KL Levine M Nelson EJ . Etiology of severely dehydrating diarrheal illness in infants and young children residing in low- and middle-income countries. Open Forum Infect Dis. (2024) 11:ofae619. doi: 10.1093/ofid/ofae619. PMID: 39494449 PMC11530959

[6] UN News . Cholera vaccine shortage forces shift to one-dose regimen (2022). Available online at: https://news.un.org/en/story/2022/10/1129672 (Accessed January 26, 2026).

[7] UN News . Cholera outbreak in West and Central Africa poses crisis for children (2025). Available online at: https://news.un.org/en/story/2025/07/1165528 (Accessed January 26, 2026).

[8] UN News . WHO reports exponential rise in cholera cases in Africa (2023). Available online at: https://news.un.org/en/story/2023/02/1133337 (Accessed January 26, 2026).

[9] Burke RM Ramani S Lynch J Cooper LV Cho H Bandyopadhyay AS . Geographic disparities impacting oral vaccine performance: observations and future directions. Clin Exp Immunol. (2025) 219:uxae124. doi: 10.1093/cei/uxae124. PMID: 39774633 PMC11773816

[10] Xu H Tiffany A Luquero FJ Kanungo S Bwire G Qadri F . Protection from killed whole-cell cholera vaccines: a systematic review and meta-analysis. Lancet Glob Health. (2025) 13:e1203–e1212. doi: 10.1016/S2214-109X(25)00107-X. PMID: 40580988 PMC12208782

[11] Bi Q Ferreras E Pezzoli L Legros D Ivers LC Date K . Protection against cholera from killed whole-cell oral cholera vaccines: a systematic review and meta-analysis. Lancet Infect Dis. (2017) 17:1080–8. doi: 10.1016/S1473-3099(17)30359-6. PMID: 28729167 PMC5639147

[12] Chisti MJ . Killed whole-cell cholera vaccines: is evidence sufficient for their widespread use? Lancet Glob Health. (2025) 13:e1152–e1153. doi: 10.1016/S2214-109X(25)00187-1. PMID: 40580979

[13] Luquero FJ Azman AA . Protection of young children with cholera. Lancet Infect Dis. (2018) 18:947–8. doi: 10.1016/S1473-3099(18)30465-1. PMID: 30152359

[14] Ryan ET Leung DT Jensen O Weil AA Bhuiyan TR Khan AI . Systemic, mucosal, and memory immune responses following cholera. Trop Med Infect Dis. (2021) 6:192. doi: 10.3390/tropicalmed6040192. PMID: 34842841 PMC8628923

[15] Clements JD Norton EB . The mucosal vaccine adjuvant LT(R192G/L211A) or dmLT. mSphere. (2018) 4:e00215–e00218. doi: 10.1128/mSphere.00215-18. PMID: 30045966 PMC6060342

[16] Crothers JW Norton EB . Recent advances in enterotoxin vaccine adjuvants. Curr Opin Immunol. (2023) 85:102398. doi: 10.1016/j.coi.2023.102398. PMID: 37976963 PMC11258862

[17] Maciel M Scott JC Baudier RL Clements JD Laird RM Gutiérrez RL . Protective antibodies against enterotoxigenic Escherichia coli are generated from heat-labile toxoid vaccination and exhibit subject- and vaccine-specific diversity. Med Microbiol Immunol. (2025) 214:10. doi: 10.1007/s00430-025-00817-3. PMID: 39934422 PMC11814043

[18] Larena M Holmgren J Lebens M Terrinoni M Lundgren A . Cholera toxin and related nontoxic adjuvants mmCT and dmLT, promote human Th17 responses via cyclic AMP-protein kinase A and inflammasome-dependent IL-1 signaling. J Immunol. (2015) 194:3829–39. doi: 10.4049/jimmunol.1401633. PMID: 25786687

[19] El-Kamary SS Cohen MB Bourgeois AL Van De Verg L Bauers N Reymann M . Safety and immunogenicity of a single oral dose of recombinant double mutant heat-labile toxin derived from enterotoxigenic Escherichia coli. Clin Vaccine Immunol. (2013) 20:1764–70. doi: 10.1128/CVI.00464-13. PMID: 24049109 PMC3837789

[20] Bhuiyan TR Khanam F Basher SR Dash P Chowdhury MI Haque S . Safety and immunogenicity of a recombinant double-mutant heat-labile toxin derived from enterotoxigenic Escherichia coli in healthy Bangladeshi adults delivered by three different routes. Front Bacteriol. (2025) 4:1567791. doi: 10.3389/fbrio.2025.1567791. PMID: 41306922 PMC12646041

[21] Alam MM Riyadh MA Fatema K Rahman MA Akhtar N Qadri F . Antigen-specific memory B-cell responses in Bangladeshi adults after one- or two-dose oral killed cholera vaccination and comparison with responses in patients with naturally acquired cholera. Clin Vaccine Immunol. (2011) 18:844–50. doi: 10.1128/CVI.00562-10. PMID: 21346055 PMC3122537

[22] Arifuzzaman M Rashu R Leung DT Hosen MI Bhuiyan TR Bhuiyan MR . Antigen-specific memory T-cell responses after vaccination with an oral killed cholera vaccine in Bangladeshi children and comparison to responses in patients with naturally acquired cholera. Clin Vaccine Immunol. (2012) 19:1304–11. doi: 10.1128/CVI.00196-12. PMID: 22739692 PMC3416086

[23] Chowdhury F Begum YA Alam MM Khan AI Ahmed T Bhuiyan MR . Concomitant enterotoxigenic Escherichia coli infection induces increased immune responses to Vibrio cholerae O1 antigens in patients with cholera in Bangladesh. Infect Immun. (2010) 78:2117–24. doi: 10.1128/IAI.01426-09. PMID: 20176796 PMC2863502

[24] Rollwagen FM Pacheco ND Clements JD Pavlovskis O Rollins DM Walker RI . Killed Campylobacter elicits immune response and protection when administered with an oral adjuvant. Vaccine. (1993) 13:1316–22. doi: 10.1016/0264-410x(93)90101-3. PMID: 8296484

[25] Clements JD Finkelstein RA . Isolation and characterization of homogeneous heat-labile enterotoxins with high specific activity from Escherichia coli cultures. Infect Immun. (1979) 24:760–9. doi: 10.1128/iai.24.3.760-769.1979. PMID: 89088 PMC414371

[26] Sanchez JL Trofa AF Taylor FN Kuschner RA Defraites RF Craig SC . Safety and immunogenicity of the oral, whole cell/recombinant B subunit cholera vaccine in North American volunteers. J Infect Dis. (1993) 167:1446–9. doi: 10.1093/infdis/167.6.1446. PMID: 8501336

[27] Jertborn M Svennerholm AM Holmgren J . Safety and immunogenicity of an oral recombinant cholera B subunit-whole cell vaccine in Swedish volunteers. Vaccine. (1992) 10:130–2. doi: 10.1016/0264-410x(92)90030-n. PMID: 1539466

[28] Qadri F Ryan ET Faruque AS Ahmed F Khan AI Islam MM . Antigen-specific immunoglobulin A antibodies secreted from circulating B cells are an effective marker for recent local immune responses in patients with cholera: comparison to antibody-secreting cell responses and other immunological markers. Infect Immun. (2003) 71:4808–14. doi: 10.1128/IAI.71.8.4808-4814.2003. PMID: 12874365 PMC165990

[29] Rahman A Rashu R Bhuiyan TR Chowdhury F Khan AI Islam MM . Antibody-secreting cell responses after Vibrio cholerae O1 infection and oral cholera vaccination in adults in Bangladesh. Clin Vaccine Immunol. (2013) 20:1592–8. doi: 10.1128/CVI.00347-13. PMID: 23945156 PMC3807192

[30] Ali M Emch M Park JK Yunus M Clemens J . Natural cholera infection-derived immunity in an endemic setting. J Infect Dis. (2011) 204:912–8. doi: 10.1093/infdis/jir416. PMID: 21849288 PMC3156915

[31] Paisie TK Cash MN Tagliamonte MS Ali A Morris JG Salemi M . Molecular basis of the toxigenic Vibrio cholerae O1 serotype switch from Ogawa to Inaba in Haiti. Microbiol Spectr. (2023) 11:e03624-22. doi: 10.1128/spectrum.03624-22. PMID: 36537825 PMC9927444

[32] Su-Arehawaratana P Singharaj P Taylor DN Hoge C Trofa A Sadoff J . Safety and immunogenicity of different immunization regimens of CVD 103-HgR live oral cholera vaccine in soldiers and civilians in Thailand. J Infect Dis. (1992) 165:1042–8. doi: 10.1093/infdis/165.6.1042. PMID: 1583321

[33] Wiens KH Iyer AS Bhuiyan TR Lu LL Cizmeci D Gorman MJ . Predicting Vibrio cholerae infection and symptomatic disease: a systems serology study. Lancet Microbe. (2023) 4:e228–e235. doi: 10.1016/S26666-5247(22)00391-3. PMID: 36907197 PMC10186354

[34] Norton EB Lawson LB Freytag LC Clements JD . Characterization of a mutant Escherichia coli heat-labile toxin LT(R192G/L211A), as a safe and effective oral adjuvant. Clin Vaccine Immunol. (2011) 18:546–51. doi: 10.1128/CVI.00538-10. PMID: 21288994 PMC3122563

[35] Alam MM Bufano MK Xu P Kalsy A Yu Y Freeman YW . Evaluation in mice of a conjugate vaccine for cholera made from Vibrio cholerae O1 (Ogawa) O-specific polysaccharide. PloS NeglTrop Dis. (2014) 8:e2683. doi: 10.1371/journal.pntd.0002683. PMID: 24516685 PMC3916310

[36] Upadhyay I Li S Ptacek G Seo H Sack DA Zhang W . A polyvalent multiepitope protein cross-protects against Vibrio cholerae infection in rabbit colonization and passive protection models. Proc Natl Acad Sci USA. (2022) 119:e2202938119. doi: 10.1073/pnas.2202938119. PMID: 36469767 PMC9897427

[37] Walker RI Bourgeois AL . Oral inactivated whole cell vaccine for mucosal immunization: ETVAX case study. Front Immunol. (2023) 14:1125102. doi: 10.3389/fimmu.2023.1125102. PMID: 36936951 PMC10018008

[38] Kantele A Riekkinen M Jokiranta TS Pakkanen SH Pietilä JP Svennerholm AM . Safety and immunogenicity of ETVAX, an oral inactivated vaccine against enterotoxigenic Escherichia coli diarrhea: a double-blinded, randomized, placebo-controlled trial among Finnish travellers to Benin, West Africa. J Travel Med. (2023) 30:taad045. doi: 10.1093/jtm/taad045. PMID: 37099803 PMC10658657

[39] Qadri F Akhtar M Bhuiyan TR Chowdhury MI Ahmed T Rafique TA . Safety and immunogenicity of the oral, inactivated, enterotoxigenic Escherichia coli vaccine ETVAX in Bangladeshi children and infants: a double-blind, randomized, placebo-controlled phase 1/2 trial. Lancet Infect Dis. (2020) 20:208–19. doi: 10.1016/S1473-3099(19)30571-7. PMID: 31757774 PMC6990395

[40] Svennerholm AM Lundgren A Leach S Akhtar M Qadri F . Mucosal immune responses against an oral enterotoxigenic Escherichia coli vaccine evaluated in clinical trials. J Infect Dis. (2021) 224:S821–S828. doi: 10.1093/infdis/jiab475. PMID: 34550392 PMC8687049

[41] Tobias J Holmgren J Hellman M Nygren E Lebens M Svennerholm AM . Over-expression of major colonization factors of enterotoxigenic Escherichia coli, alone or together, on non-toxigenic E. coli bacteria. Vaccine. (2010) 28:6977–84. doi: 10.1016/j.vaccine.2010.08.047. PMID: 20728524

[42] Lebens M Shahabi V Bäckstrom M Houze T Lindblad M Holmgren J . Synthesis of hybrid molecules between heat-labile enterotoxin and cholera toxin B subunits: potential for use in a broad-spectrum vaccine. Infect Immun. (1996) 64:2144–50. doi: 10.1128/iai.64.6.2144-2150.1996. PMID: 8675319 PMC174048

[43] Sukwa N Mubanga C Hatyoka LM Chilyabanyama ON Chibuye M Mundia S . Safety, tolerability and immunogenicity of an oral inactivated ETEC vaccine (ETVAX) with dmLT adjuvant in healthy adults and children in Zambia: an age descending randomised, placebo-controlled trial. Vaccine. (2023) 41:6884–94. doi: 10.1016/j.vaccine.2023.09.052. PMID: 37838479

[44] Mubanga C Simuyandi M Mwape K Chibesa K Chisenga C Chilyabanyama ON . Use of an ETEC proteome microarray to evaluate cross-reactivity of ETVAX vaccine-induced IgG antibodies in Zambian children. Vaccines. (2023) 11:939. doi: 10.3390/vaccines11050939. PMID: 37243042 PMC10224314

[45] Hossain J Secka F Sanyang C Raifu T Okoh EC Olubiyi OA . Results of a phase 2B double-blind, randomised, controlled efficacy trial of ETVAX, a vaccine against enterotoxigenic E. coli-positive diarrhoea in Gambian children. Lancet Infect Dis. (2026). doi: 10.1016/s1473-3099(25)00774-1. PMID: 41713476 PMC13241577

[46] Svennerholm AM Qadri F Lundgren A Kaim J Bhuiyan TR Akhtar M . Induction of mucosal and systemic immune responses against the common O78 antigen of an oral inactivated ETEC vaccine in Bangladeshi children and infants. Vaccine. (2022) 40:380–9. doi: 10.1016/j.vaccine.2021.10.056. PMID: 34772542

[47] Mottram L Lundgren A Svennerholm AM Leach S . A systems biology approach identifies B cell maturation antigen (BCMA) as a biomarker reflecting oral vaccine induced IgA antibody responses in humans. Front Immunol. (2021) 12:647873. doi: 10.3389/fimmu.2021.647873. PMID: 33828557 PMC8019727

[48] Akhtar M Nizam NN Basher SR Hossain L Akter S Bhuiyan TR . dmLT adjuvant enhances cytokine responses to T cell stimuli, whole cell vaccine antigens and lipopolysaccharide in both adults and infants. Front Immunol. (2021) 12:654872. doi: 10.3389/fimmu.2021.654872. PMID: 34054818 PMC8160295

[49] Levine MM . Immunogenicity and efficacy of oral vaccines in developing countries: lessons learned from a live oral cholera vaccine. BMC Biol. (2010) 8:129. doi: 10.1186/1741-7007-8-129. PMID: 20920375 PMC2958895

[50] Fakoya B Sit B Waldor MK . Transient intestinal colonization by a live-attenuated oral cholera vaccine induces protective immune responses in streptomycin-treated mice. J Bacteriol. (2020) 202:e00232-20. doi: 10.1128/JB.00232-20. PMID: 32540930 PMC7685556

[51] Chen WH Cohen MB Kirkpatrick BD Brady RC Galloway D Gurwith M . Single-dose live oral cholera vaccine CVD 103-HgR protects against human experimental infection with Vibrio cholerae O1 El Tor. Clin Infect Dis. (2016) 62:1329–35. doi: 10.1093/cid/ciw145. PMID: 27001804 PMC4872293

[52] Harro C Bourgeois AL Sack D Walker R DeNearing B Brubaker J . Live attenuated enterotoxigenic Escherichia coli (ETEC) vaccine with dmLT adjuvant protects human volunteers against virulent experimental ETEC challenge. Vaccine. (2019) 37:1978–86. doi: 10.1016/j.vaccine.2019.02.025. PMID: 30797634 PMC6434318

[53] Feinmann J . Vaccine shortages worsen the deadliest cholera outbreaks in years. BMJ. (2024) 385:q1228. doi: 10.1136/bmj.q1228. PMID: 38889931

